# Wellens syndrome during chemotherapy for cholangiocarcinoma: A case report of cardiovascular toxicity associated with gemcitabine-containing regimen

**DOI:** 10.1097/MD.0000000000033599

**Published:** 2023-04-28

**Authors:** Xufei Liang, Xuhong Geng, Xiaohong Gong, Xi Yin, Yongzhen Chen

**Affiliations:** a Department of Function, Fourth Hospital of Hebei Medical University, Shijiazhuang, China.

**Keywords:** acute coronary syndrome, chemotherapy-related cardiovascular toxicity, cholangiocarcinoma, wellens syndrome

## Abstract

**Rationale:**

Wellens syndrome is a comprehensive electrocardiographic (ECG) diagnosis that combines medical history with characteristic ECG changes. These changes, characterized by biphasic T-wave inversions or symmetric and deep T-wave inversions in the anterior precordial leads, often indicate that the left anterior descending coronary artery is at a high risk of severe stenosis. Chemotherapy-related cardiovascular toxicity refers to damage to the cardiovascular system caused by chemotherapeutic drugs, which is unpredictable and may occur during or after chemotherapy.

**Patient concerns:**

In this case report, a 41-year-old male patient with cholangiocarcinoma received sequential adjuvant chemotherapy with gemcitabine/nanoparticle albumin–bound paclitaxel and gemcitabine/cisplatin. This patient presented with recurrent brief chest pain episodes after the third dose of gemcitabine/cisplatin, and the characteristic T-wave morphological changes were captured in routine ECG monitoring prior to the 6th dose.

**Diagnoses:**

Acute coronary syndrome due to chemotherapy-related cardiovascular toxicity was diagnosed on the basis of characteristic ECG changes.

**Interventions:**

The patient underwent coronary angiography, which revealed diffuse stenosis of up to 95% in the middle segment of the left anterior descending coronary artery. Stents were implanted in the stenotic segment for vascular reconstruction.

**Outcomes:**

The patient’s chest pain was completely resolved, and electrocardiography returned to normal.

**Lessons:**

Cardiovascular toxicity during chemotherapy in patients with cancer may be life threatening. This rare case highlights the importance of identifying the characteristic ECG pattern of the Wellens syndrome by monitoring electrocardiography during chemotherapy. Immediate and accurate identification of the morphological ECG features of Wellens syndrome with a slight elevation of the ST-segment is related to patient prognosis.

## 1. Introduction

Most anticancer therapies have a wide range of short- and long-term cardiotoxic effects ranging from asymptomatic and transient to clinically relevant and long-lasting cardiac events.^[1]^ However, after weighing the potential cardiovascular damage resulting from anticancer chemotherapy against its potential benefits, cardiac monitoring during chemotherapy may be a good option. A large amount of evidence indicates that cardiac monitoring in certain anticancer settings limits the adverse effects on the cardiovascular system of patients.^[2,3]^ Wellens syndrome is an electrocardiographic (ECG) diagnosis initially characterized by biphasic T-wave inversions, and later by symmetric or deep T-wave inversions in the anterior precordial leads. In patients with a history of angina pectoris, Wellens syndrome is closely associated with severe stenosis and critical obstruction of the left anterior descending coronary artery (LADCA).^[4–6]^ This case presented here is a rare case of acute coronary syndrome (ACS) resulting from chemotherapy-related cardiovascular toxicity (CTRCVT) during adjuvant chemotherapy in a patient with cholangiocarcinoma revealed by the ECG pattern of Wellens syndrome.

## 2. Case presentation

**Figure 1.**
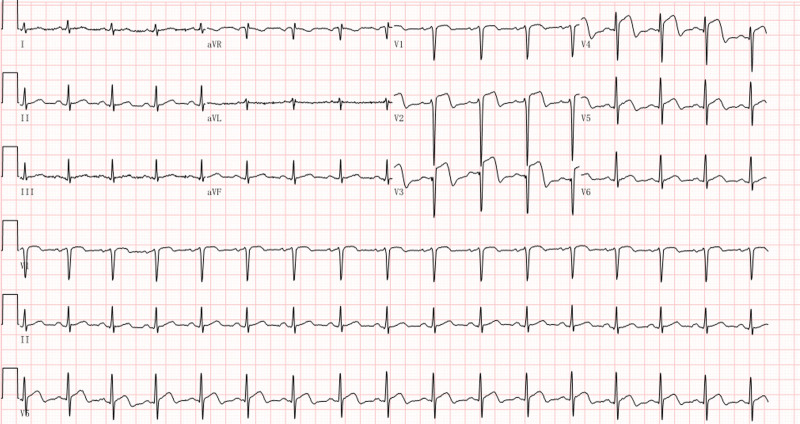
Initial findings of routine Electrocardiogram: A heart rate of 99 beats per minute (bpm); sinus rhythm; ST-segment elevation in V2 to V4 leads with an oblique straight upward shape and biphasic T-wave in V2 through V5 leads; a mild QTc prolongation of 479 ms. QTc = QT interval.

**Figure 2.**
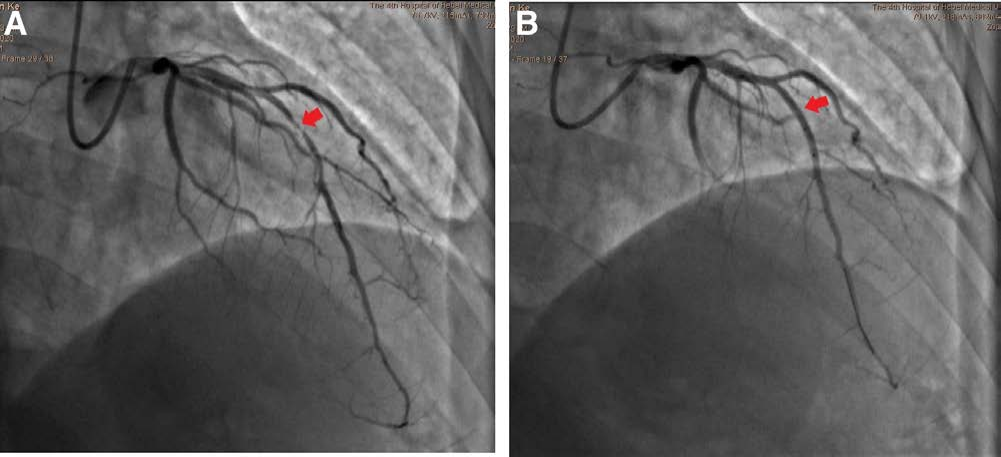
Coronary angiography findings of pre-(A) and post-vascular reconstruction (B): (A) the red arrow points to the diffuse stenosis of the middle segment of the left anterior descending coronary artery. (B) This segment of the left anterior descending coronary artery achieved reperfusion after vascular reconstruction.

**Figure 3.**
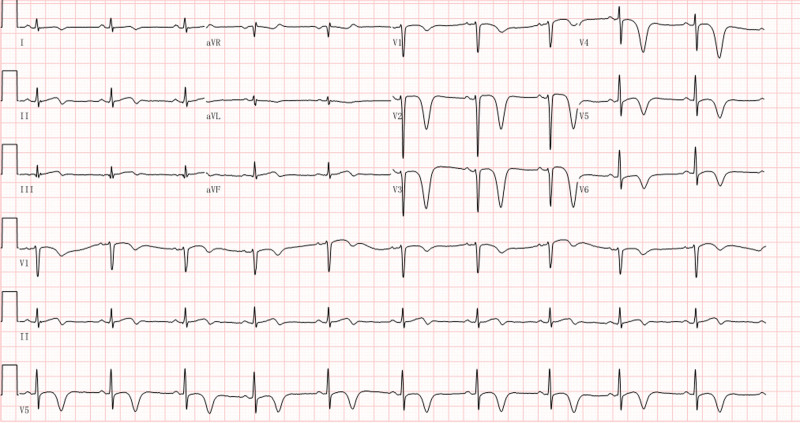
Electrocardiogram findings after vascular reconstruction: A heart rate of 61 bpm; sinus rhythm; deeply inverted T waves in leads V2 through V6 leads.

A 41-year-old male was admitted to our hospital because of a space-occupying liver lesion that was found on physical examination. The patient had no history of cardiovascular diseases. Routine admission did not reveal any obvious positive findings. The space-occupying lesion in the left lobe of the liver was successfully resected and eventually confirmed by pathology as a cholangiocarcinoma. After a stable perioperative period of approximately 40 days, intravenous infusion of gemcitabine/nanoparticle albumin–bound paclitaxel (nab-paclitaxel) combined with adjuvant chemotherapy was initiated. Chemotherapy had to be discontinued after applying 6 doses owing to the patient’s intolerance to leg numbness and was resumed with a combined regimen of gemcitabine/cisplatin. The patient developed intermittent temporary chest distress after the third dose of gemcitabine/cisplatin. No synchronous abnormalities were observed on the ECG. As the treatment progressed, the precordial discomfort gradually aggravated into intermittent episodes of substernal pain lasting for several minutes. The routine 12-lead electrocardiogram of the patient, who did not complain of any discomfort, was immediately referred to us before the 6th dose of gemcitabine/cisplatin infusion (Fig. 1). ST-segment elevation appeared in leads V2 to V4, with an oblique straight upward shape, and the biphasic T-wave appeared in leads V2 to V5 with a corrected QT interval of 479 ms. An electrocardiogram with characteristic biphasic T-wave changes and an intermittent history of angina pectoris reported by the patient suggested that this patient had a high risk of impending LADCA (LADCA) occlusion. This suggestion was confirmed by a significant increase in serum cardiac markers several hours after the patient was referred to the cardiology department for emergency treatment. The peak concentrations of cardiac troponin I and N-terminal prohormone of brain natriuretic peptide were as high as 19.6 ng/mL (normal value < 0.1 ng/mL) and 2980.0 pg/mL (normal < 125 pg/mL), respectively. Emergency coronary angiography showed diffuse stenosis in the middle segment of the LADCA, which was up to 95% severe (Fig. 2 A). This problem was resolved by stent implantation (Fig. 2 B). After vascular reconstruction, the patient’s precordial discomfort disappeared and his ECG pattern returned to normal (Fig. 3). It should be noted that T-wave inversion in the anterior precordial leads on this electrocardiogram is a normal performance that can be observed during the recovery period of the LADCA to achieve reperfusion.^[7]^ The patient did not complete the remaining adjuvant chemotherapy to avoid a possible secondary cardiac event and was successfully discharged.

## 3. Discussion

This is a rare report of ACS events induced by cardiovascular toxicity during adjuvant chemotherapy in a patient with cholangiocarcinoma clinically diagnosed as having Wellens syndrome by electrocardiography. In 1982, Wellens et al described for the first time that a subgroup of patients admitted for unstable angina had a high risk of progressing to anterior wall myocardial infarction (MI) and that they all had similar characteristic ECG patterns of changes in the anterior precordial leads, which involved T-wave inversions and occasionally the ST-segment.^[4]^ Soon after, this finding was confirmed by other studies.^[5]^ Wellens syndrome is a characteristic ECG pattern of changes that can predict impending severe stenosis and occlusion of the LADCA.^[6]^ The criteria for Wellens syndrome include: Biphasic T-wave inversions (initially positive followed by negative deflections from baseline) and symmetric or deep T-wave inversions in leads V2 to V3, but sometimes found in V1, V4, V5, and V6; Minimal or no ST-segment elevation (<1 mm); No precordial Q waves or loss of R waves; A prior history of angina pectoris; The presence of these ECG features when the patient is free of chest pain; and Normal to slightly elevated cardiac markers.

In clinical settings, the diagnosis of Wellens syndrome is critical, but challenging.^[8]^ The reality is that T-wave inversions have attracted enough attention in the clinic, but biphasic T-wave inversion does not play its full predictive value and is easily ignored by clinicians. This kind of biphasic T-wave change from V2 to V3 leads to Wellens syndrome manifesting as follows: a sharp downsloping occurs after the oblique straight upsloping and ends with the formation of an inverted T-wave. The angle formed by the ascending and descending branches of the T-wave was between 60 to 90 degrees. This feature is different from the inverted T-wave shape resulting from other reasons.^[9]^ The characteristic biphasic T-wave pattern on the electrocardiogram of the patient in this case report meets the above characteristics and the diagnostic criteria of Wellens syndrome, which indicates that this patient is at a high risk of impending anterior wall MI. Accurate identification and timely diagnosis of Wellens syndrome have a positive contribution to both the assessment of coronary artery disease progression and the subsequent clinical decision-making process, especially for patients who are in a stable condition during chemotherapy. The typical abnormal changes in the ECG pattern caused by Wellens syndrome usually last for a very short period, during which the ST-segment of the leads is at the equipotential line. ST-segment elevation may occur with progression of ischemia.^[9]^ The electrocardiogram of the patient presented in this case report showed different levels of ST-segment elevation in the anterior precordial leads, especially in V2 and V3 leads (0.25 mV and 0.4 mV, respectively). However, it may not be possible to prevent anterior wall MI caused by the occlusion of the LADCA in time if this case is not diagnosed as Wellens syndrome and instead treated as ST-segment elevation myocardial ischemia, resulting in irreversible consequences.^[10]^

Chemotherapeutic drugs may jeopardize the cardiovascular system to a certain extent during tumor treatment.^[11]^ The CTRCVT has a wide range of manifestations, ranging from mild events, such as changes in blood pressure, asymptomatic ECG, and pericarditis, to more severe or fatal events, such as arrhythmia, myocarditis, cardiomyopathy, and ACS.^[12]^ The occurrence of cardiovascular toxicity varied significantly among different administration methods. Continuous intravenous administration may increase the risk of cardiovascular toxicity.^[13]^ The key drug in both regimens is gemcitabine, which is a nucleoside analog that can incorporate into deoxyribonucleic acid (DNA), terminate DNA strand elongation, and impair DNA repair by inhibiting ribonucleotide reductase.^[14]^ Gemcitabine is a relatively well-tolerated cytotoxic drug with a very low cardiac toxicity. The most common adverse side effect is dose-limiting myelosuppression.^[12]^ It is widely used to treat multiple cancers either alone or in combination. However, some studies have reported that gemcitabine may be associated with cardiovascular adverse reactions.^[13]^ It has been reported that the range of potentially lethal gemcitabine cardiac events includes myocardial ischemia, pericardial disease, supraventricular arrhythmia and heart failure.^[9]^ However, the combination regimen of gemcitabine and paclitaxel or cisplatin may exacerbate the burden on the cardiovascular system of patients with tumors because of the synergistic or superimposed effects between these drugs. A pharmacovigilance study found that in 119 reports of MI, combination therapy (gemcitabine and at least 1 other anticancer drug) was more toxic than gemcitabine alone (86/119, 72% *vs* 33/119, 28%). The proportions of cisplatin and paclitaxel in these combination regimens ranked first and second, respectively (46 and 24%, respectively). MI is the most common cardiovascular toxicity associated with the combination of gemcitabine and cisplatin. Cardiovascular toxicity due to MI accounts for the highest proportion of cardiovascular toxicity among patients treated with a combination regimen of gemcitabine and cisplatin.^[13]^ Although the dose of each drug administered may be safe and each treatment has a different mechanism of action, they may have toxic synergy or multiple strike mechanisms leading to severe cardiac toxicity.^[15,16]^

The patient’s long-term exposure to various chemotherapy drugs and the progression of all the above conditions reported in this case suggest that this was an acute episode of CTRCVT during chemotherapy. However, CTRCVT did not worsen owing to its timely detection and effective treatment, which was successful in the case presented here. To the best of our knowledge, this is probably the first report of an ACS event caused by an acute episode of cardiovascular toxicity during adjuvant chemotherapy, revealed by the key diagnostic features of Wellens syndrome. Although lethal acute cardiovascular toxicity occurred during treatment with the gemcitabine/cisplatin combination regimen, all 3 chemotherapeutic drugs with potential cardiovascular toxicity used in this case may have played a role regardless of gemcitabine,^[13]^ nab-paclitaxel^[17]^ or cisplatin.^[18]^ There are relatively few reports on ACS induced by gemcitabine-containing regimens. Several factors related to gemcitabine (dose and infusion scheme) and patient characteristics (age, electrolyte imbalance, heart disease history, and simultaneous use of other cardiotoxic drugs) can affect the incidence of gemcitabine treatment-related ACS.^[19]^ However, the specific mechanisms underlying gemcitabine-induced ACS remain unclear. However, the hypothesis that it is related to a direct injury to the endothelium or drug-induced coronary spasms is generally accepted.^[8]^ In this report, we describe a 41-year-old male patient with no history of cardiovascular disease, who developed cardiovascular toxicity in the form of ACS, which was associated with a combination regimen of gemcitabine/nab-paclitaxel and/or gemcitabine/cisplatin during chemotherapy. Through timely and accurate identification of the characteristic ECG pattern of the Wellens syndrome during routine ECG monitoring following appropriate clinical intervention, the patient’s prognosis was significantly improved. Therefore, careful and regular routine ECG examinations are necessary during the administration of gemcitabine (via intravenous infusions). This case report not only provides a reference for the careful administration of a gemcitabine-containing combination chemotherapy regimen but also provides a reference for further understanding of the cardiovascular toxicity associated with gemcitabine-containing combination chemotherapy.

## 4. Conclusion

For clinicians, accurate and timely identification of ECG features of Wellens syndrome in patients with mild ST-segment elevation in the anterior precordial leads is closely related to patient prognosis. In addition, the diagnosis of Wellens syndrome requires comprehensive and dynamic consideration of the patients individual conditions and disease progression. The risk of an acute episode of cardiovascular toxicity, including ACS, during treatment with sequential combination chemotherapy regimens containing gemcitabine does exist and may even endanger their lives. Routine ECG monitoring of cancer patients during chemotherapy is indispensable. The recognition and interpretation of characteristic ECG patterns are crucial for patients with acute episodes of cardiovascular toxicity induced by chemotherapy drugs.

## Author contributions

**Conceptualization:** Xufei Liang, Xuhong Geng, Yongzhen Chen.

**Data curation:** Xuhong Geng, Xiaohong Gong

**Formal analysis:** Xufei Liang, Xuhong Geng, Xiaohong Gong.

**Project administration:** Xuhong Geng, Xiaohong Gong, Xi Yin.

**Resources:** Xi Yin, Yongzhen Chen.

**Software:** Xufei Liang.

**Supervision:** Yongzhen Chen.

**Validation:** Xuhong Geng, Yongzhen Chen.

**Visualization:** Xufei Liang, Xi Yin.

**Writing – original draft:** Xufei Liang, Xuhong Geng, Yongzhen Chen

**Writing – review & editing:** Xufei Liang, Yongzhen Chen
